# Supplementary material for: Content-rich biological network constructed by mining PubMed abstracts
Source: BMC Bioinformatics. 2004 Oct 8;5:147. doi: 10.1186/1471-2105-5-147 (PMC528731; doi:10.1186/1471-2105-5-147)
Supplement: Additional File 2 — The original results of the above study (non-essential files are deleted to keep the file size under the limit set by BMC bioinformatics). [file 1471-2105-5-147-S2.bz2 › chilibotAdditionalFile2/dip40/501ID7878020E10667/html/TGFBR1_TGFBR2.html]

 


 **TGFBR1** and **TGFBR2** 
  
Found 40 abstracts in PubMed, retrieved 40.  
 

 What does Google say? 
 PDF only 
| .edu only 

---

**Interactive relationship** (e.g. stimulation, inhibition, etc)

**Neutral relationship**- Cloning of a TGF beta type I receptor  [ **TGFBR1** ]  that forms a heteromeric complex with the TGF beta type II receptor  [ **TGFBR2** ] .  Ref: 8242743 Cell, 1993
- Recent studies have revealed that upon TGF beta binding several serine and threonine residues in the GS domain of TGF beta type I receptor  [ **TGFBR1** ]  T beta R I are phosphorylated by TGF beta type II receptor  [ **TGFBR2** ]  T beta R II.  Ref: 8576253 J Biol Chem, 1996
- Furthermore, competition based quantitative reverse transcription PCR using synthetic internal standards revealed that leiomyomata express a significantly higher number copies per cell of TGF beta type II receptor  [ **TGFBR2** ]  mRNA, followed by TGF beta 1, TGF beta type I receptor  [ **TGFBR1** ] , TGF beta 2, and TGF beta 3 P <.  Ref: 8784073 J Clin Endocrinol Metab, 1996
- Initiation of signaling requires binding of TGF beta1 to TGF beta type II receptor  [ **TGFBR2** ]  TbetaR II, a constitutively active serine threonine kinase, which subsequently transphosphorylates TGF beta type I receptor  [ **TGFBR1** ]  TbetaR I.  Ref: 10997691 Kidney Int Suppl, 2000
- We generated two different chimeric TGF beta superfamily receptors, i.e. TbetaR I BMPR IB, containing the extracellular domain of TGF beta type I receptor  [ **TGFBR1** ]  TbetaR I and the intracellular domain of bone morphogenetic protein type IB receptor BMPR IB, and TbetaR II ActR IIB, containing the extracellular domain of TGF beta type II receptor  [ **TGFBR2** ]  TbetaR II and the intracellular domain of activin type IIB receptor ActR IIB.  Ref: 9261125 J Biol Chem, 1997

**Non-interactive relationship** (e.g. studied together, co-existance, homology, etc.)

- No mutations of ALK5  [ **TGFBR1** ]  or  **TGFBR2**  were found.  Ref: 11408925 Int J Oncol, 2001
- METHODS The mRNA expression of TGF beta1, TGF beta type I receptor  [ **TGFBR1** ]  TGFbetaRI, TGF beta type II receptor  [ **TGFBR2** ]  TGFbetaRII and TGF beta type III receptor TGFbetaRIII in rat livers injured by CCl4 administration was studied by Northern blotting.  Ref: 9566825 J Hepatol, 1998
- We therefore studied the expression of UPA, UPAR, IGF2R, ALK5  [ **TGFBR1** ] ,  **TGFBR2** , TGFBR3, ENG, ALK1, TGFB1, TGFB2, and TGFB3 in a series of 14 pancreatic carcinoma cell lines.  Ref: 11408925 Int J Oncol, 2001
- However, we observed a markedly low TGF beta type I receptor  [ **TGFBR1** ]  protein level, while the malignant endometrium showed a significantly higher TGF beta type II receptor  [ **TGFBR2** ]  protein level than the normal endometrium.  Ref: 12213293 Cancer Lett, 2002
- These genes were TGF beta type I receptor  [ **TGFBR1** ]  gene  **TGFBR1** , TGF beta type II receptor  [ **TGFBR2** ]  gene  **TGFBR2** , SMAD2 gene SMAD2, SMAD3 gene SMAD3, SMAD4 gene SMAD4, and SMAD7 gene SMAD7, all of which compose the TGF beta1 signaling pathway.  Ref: 12202987 J Hum Genet, 2002
- In the present study we have analyzed and compared, by immunohistochemistry and in situ hybridization, the expression pattern of the R4 ALK5  [ **TGFBR1** ]  transforming growth factor TGF beta type I receptor  [ **TGFBR1** ]  RI and the TGF beta type II receptor  [ **TGFBR2** ]  RII in normal human skin, in wounded skin at various stages during the transition of wound granulation tissue to scar, and in long persisting post burn hypertrophic scars.  Ref: 9466575 Am J Pathol, 1998
- METHOD To determine the roles of the transforming growth factor beta 1 TGF beta 1, and TGF beta type I receptor  [ **TGFBR1** ]  T beta R I, and the TGF beta type II receptor  [ **TGFBR2** ]  T beta R II in the progression of a pulmonary adenocarcinoma, their respective expressions have been immunohistologically studied in specimens from 120 pulmonary adenocarcinoma patients.  Ref: 9142180 J Surg Oncol, 1997
- These results suggest that the ALK5  [ **TGFBR1** ]  SMAD4 part of the TGFB signalling pathway is a major target for inactivation in pancreatic carcinomas, that the expression of  **TGFBR2** , TGFBR3, and receptors involved in TGFB activation are maintained,.  Ref: 11408925 Int J Oncol, 2001
- We demonstrate that primary cultured human thymic epithelial cells TEC express TGF beta 1, TGF beta 2 and TGF beta 3, as well as TGF beta type I receptor  [ **TGFBR1** ]  T beta RI ALK 5  [ **TGFBR1** ]  and TGF beta type II receptor  [ **TGFBR2** ]  T beta RII transcripts.  Ref: 8562514 Int Immunol, 1995
- In this study we have investigated the expression of the precursor forms of TGF beta1, TGF beta2, TGF beta3, as well as TGF beta type I receptor  [ **TGFBR1** ]  TGF betaRI and TGF beta type II receptor  [ **TGFBR2** ]  TGF betaRII in the synovial tissue of arthritic rats during the course of the disease.  Ref: 9010265 Clin Exp Immunol, 1997
- We generated mice expressing an activated TGF beta type I receptor  [ **TGFBR1** ]  or dominant negative TGF beta type II receptor  [ **TGFBR2** ]  under control of the mouse mammary tumor virus promoter.  Ref: 12808151 Proc Natl Acad Sci U S A< MedlineTA>Proc Natl Acad Sci U S A, 1997
- Eighty two % of these pancreatic cancers had genetic inactivation of the DPC4, p15, ALK 5  [ **TGFBR1** ] , or  **TGFBR2**  genes.  Ref: 9850059 Cancer Res, 1998
- We also analysed ALK5  [ **TGFBR1** ]  and  **TGFBR2**  for mutations, cell surface localisation of  **TGFBR2**  and ENG, and TGFB1 response.  Ref: 11408925 Int J Oncol, 2001
- We compared the expression of mRNA for alpha 1 I collagen, TGF beta, TGF beta type I receptor  [ **TGFBR1** ]  and TGF beta type II receptor  [ **TGFBR2** ]  in early and late passage fibroblasts by Northern blot hybridizations.  Ref: 9558492 Arch Dermatol Res, 1998
- Polyamine depletion by DFMO increased levels of the TGF beta type I receptor  [ **TGFBR1** ]  mRNA and protein but had no effect on the TGF beta type II receptor  [ **TGFBR2** ]  expression.  Ref: 11003584 Am J Physiol Cell Physiol, 2000
